# *Pvt1*-encoded microRNAs in oncogenesis

**DOI:** 10.1186/1742-4690-5-4

**Published:** 2008-01-14

**Authors:** Gabriele B Beck-Engeser, Amy M Lum, Konrad Huppi, Natasha J Caplen, Bruce B Wang, Matthias Wabl

**Affiliations:** 1 Department of Microbiology and Immunology, University of California, San Francisco, CA 94143-0414, USA; 2 Picobella, L.L.C., 863 Mitten Road, Suite 101, Burlingame, CA 94010, USA; 3 Gene Silencing Section, Genetics Branch, Center Cancer Research, National Cancer Institute, Bethesda, MD 20892, USA

## Abstract

**Background:**

The functional significance of the *Pvt1 *locus in the oncogenesis of Burkitt's lymphoma and plasmacytomas has remained a puzzle. In these tumors, *Pvt1 *is the site of reciprocal translocations to immunoglobulin loci. Although the locus encodes a number of alternative transcripts, no protein or regulatory RNA products were found. The recent identification of non-coding microRNAs encoded within the *PVT1 *region has suggested a regulatory role for this locus.

**Results:**

The mouse *Pvt1 *locus encodes several microRNAs. In mouse T cell lymphomas induced by retroviral insertions into the locus, the *Pvt1 *transcripts, and at least one of their microRNA products, mmu-miR-1204 are overexpressed. Whereas up to seven co-mutations can be found in a single tumor, in over 2,000 tumors none had insertions into both the *Myc *and *Pvt1 *loci.

**Conclusion:**

Judging from the large number of integrations into the *Pvt1 *locus – more than in the nearby *Myc *locus – *Pvt1 *and the microRNAs encoded by it are as important as *Myc *in T lymphomagenesis, and, presumably, in T cell activation. An analysis of the co-mutations in the lymphomas likely place *Pvt1 *and *Myc *into the same pathway.

## Background

Ever since its discovery in 1984 [1], the *Pvt1 *locus (in humans *PVT1*, for plasmacytoma variant translocation) has remained enigmatic. Although human and mouse *PVT1 *directs the synthesis of a large transcript, which gives rise to a variety of RNAs in normal cells [2-4], no protein product or regulatory RNA were identified. Nevertheless, the importance of the *PVT1 *locus is gleaned from the observations that it is the site of both tumorigenic translocations and retroviral insertions. In Burkitt's lymphoma, the so-called 'variant' translocations, T(2:8) or T(8:22), found in about 20% of such tumors, juxtapose immunoglobulin kappa or lambda light chain genes to the *PVT1 *locus. This results in chimeric transcripts of 0.9 to 1.2 kilobase (kb), containing the first exon of *PVT1 *on chromosome 8 and the constant region of kappa or lambda [4,5]. Although the chimeric transcripts might contribute to tumor formation, an oncogenic effect could also be mediated by the *MYC *protooncogene, just 40 to 60 kb upstream. Indeed, 80% of the translocations in Burkitt's lymphoma juxtapose *MYC *to the immunoglobulin heavy chain locus, with *MYC *being overexpressed as a consequence. Since *MYC *is also overexpressed in cells with variant translocations, it has been thought that activation of *MYC *may occur either directly [4], at a remarkable distance along the chromosome, or indirectly, via the *PVT1 *gene product [3,6].

In multiple myeloma, 16% of patients have the *PVT1 *region rearranged, but independent of the immunoglobulin loci [7]. In most murine plasmacytomas, t(15:12) translocations, analogous to the T(8:14) translocations in Burkitt's lymphoma, fuse the 5' end of the c-*Myc *gene to an immunoglobulin heavy-chain gene; there are also the t(6:15) translocations, where the chromosome 6 breakpoint is near the constant region of kappa and the chromosome 15 sequences are from the *Pvt1 *locus [1,6]. In these plasmacytomas, the expression of the (truncated) *Pvt1 *transcripts is increased [3].

*Pvt1 *is also a common retroviral integration site in murine leukemia virus (MLV) induced T lymphomas in mice [8] and rats [9,10]. Common integration sites identify protooncogenes and tumor suppressor genes, because the provirus not only acts as a mutagen, but it also "tags" the integration site with its own sequences [11]. The so-called proviral tagging method has been used to identify many new protooncogenes as well as to confirm already known protooncogenes discovered by virtue of their homology to viral oncogenes, and entire genomes have been searched for genes involved in cancer development [12-21]. These genes include non-coding RNA [22], such as oncogenic microRNAs (miRNAs) [23-25], for which models in viral oncogenesis have been described [26]. In the proviral tagging method, mice are infected with a oncogenesis have been described [26]. In the proviral tagging method, mice are infected with a retrovirus that does not contain any oncogene (for example, MLV). The virus integrates into the cellular genome and inserts its DNA near or within genes, which leads to various outcomes: (i) The insertion site is too far away from a protooncogene and thus does not activate it. In this case, there will be no selection for that cell. (ii) The provirus inserts near a protooncogene, but not within the gene (type 1). In this case, either the viral promoter, or the viral enhancer increases the expression level of the protooncogene. (iii) The provirus inserts within a gene, destroying or altering its function (type 2). In both type 1 and type 2 insertion events, if the gene is not a protooncogene or tumor suppressor gene, there will be no selection for that cell. If integration results in formation of a tumor, genomic DNA adjacent to the integration site can be recovered, sequenced and mapped to the genome. Genes neighboring the proviral integration can then be identified and classified as either protooncogenes or tumor suppressor genes.

In a large-scale retroviral insertion mutagenesis screen, we used MLV strain SL3-3, which causes T lymphomas [27]. We previously demonstrated that a group of these retroviral insertions induces overexpression of the oncogenic mmu-mir-17 miRNA cistron [23] and mmu-mir-106a [24], among other miRNAs. The *Pvt1 *locus is among the top targets of retroviral insertions in T lymphomas, but it encodes transcripts with no known protein product. Recently, *PVT1 *based miRNA candidates have been identified and confirmed experimentally [28], and here we studied the effect of MLV integration on the expression of *Pvt1 *and the miRNAs. By virtue of being tagged by the retrovirus that mediated tumor formation, these miRNAs could then be defined as oncogenic.

## Results and Discussion

### Retroviral integrations into the *Myc *and *Pvt1 *loci

**Figure 1:**
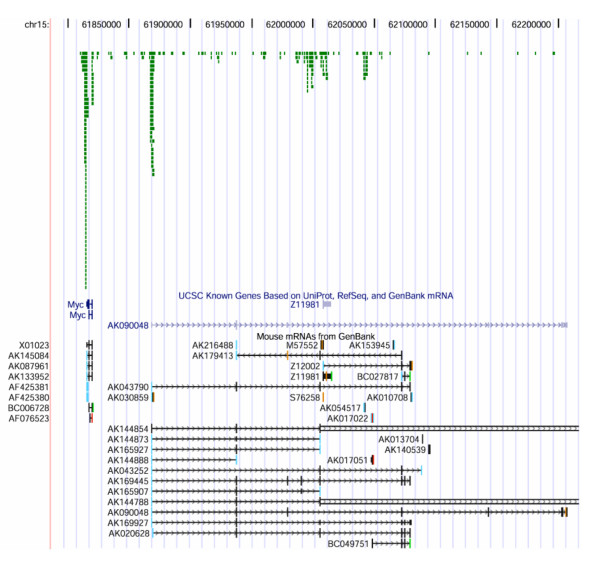
**The *Pvt1 *and the *Myc *loci have separate and distinct common integration sites**. Screen print of a customized version of the UCSC genome website browser (mouse February 2006 (mm8) genome assembly) depicting the *Myc *and the *Pvt1 *locus in the mouse. Numbers at the top, nucleotide position at chromosome 15. Green squares indicate insertion sites. Below them are the exon-intron structures of *Myc *and *Pvt1*, respectively. *Pvt1 *is represented by two reference sequences, AK090048 and Z11981, which do not share any sequence homology, but there are other transcripts as well: Below the reference sequences, there are various mouse mRNAs from GenBank. In this representation, the myc exons (thin vertical bars) are compressed. Introns are represented by horizontal lines, with the arrows denoting direction of transcription. Myc transcription is from left to right, and most Pvt1 transcripts are also from left to right.

We identified 6234 integration sites, or tags, in 2199 T-cell tumors. In these tumors, 243 tags were located at or near the *Pvt1 *locus, distributed over a region of 679,620 bp; additionally 134 tags were located at the *Myc *locus, distributed over 105,445 bp (Fig. 1). The proviral inserts were in both sense and anti-sense orientations with respect to each transcript encoded by the *Myc *and *Pvt1 *loci, respectively. The Mouse Retroviral Tagged Cancer Gene Database [29], which compiles retroviral insertions into the genomic DNA from various non-T cell derived mouse tumors, also lists 37 integrations when searched by the *Myc *locus, some of which are in fact in the *Pvt1 *locus. Insertions at the *Pvt1 *locus were originally reported in myelogenous mouse leukemia [29], and, as mentioned above, in the work defining the *Pvt1 *locus in T lymphomas induced by MLV in both mice and rats [8-10]. Remarkably, in a separate screen (data not shown) where we recovered 1798 tags from B lymphomas induced by the MLV strain Akv [15,30], only one tag was found at the *Myc *locus, and none at the *Pvt1 *locus.

Fig. 1 shows a customized screen print of the UCSC genome web site browser, looking at the *Myc *and *Pvt1 *loci. The bars in green represent the retroviral insertions in T lymphomas studied here; below them are the exon-intron structures of *Myc *and *Pvt1*, respectively. At the *Myc *locus, there are two main integration sites clusters flanking the gene upstream and downstream of it. Whereas the *Myc *transcript is clearly defined, there are several alternative transcripts depicted for *Pvt1*, a variety that was noted before [2-4]. Notably, there are two reference sequences, AK090048 and Z11981, which do not share any sequences, but are denoted as *Pvt1 *nevertheless. Furthermore, among the mRNAs from GenBank, there are other fragments of apparently intronic transcripts, and there is AK030859, which represents an extended exon 1. At any rate, there are three main integration site clusters at the *Pvt1 *locus, as represented by transcript AK090048 – one upstream of the transcript, and two within the locus.

### Transcriptional orientation of provirus and target gene

**Figure 2:**
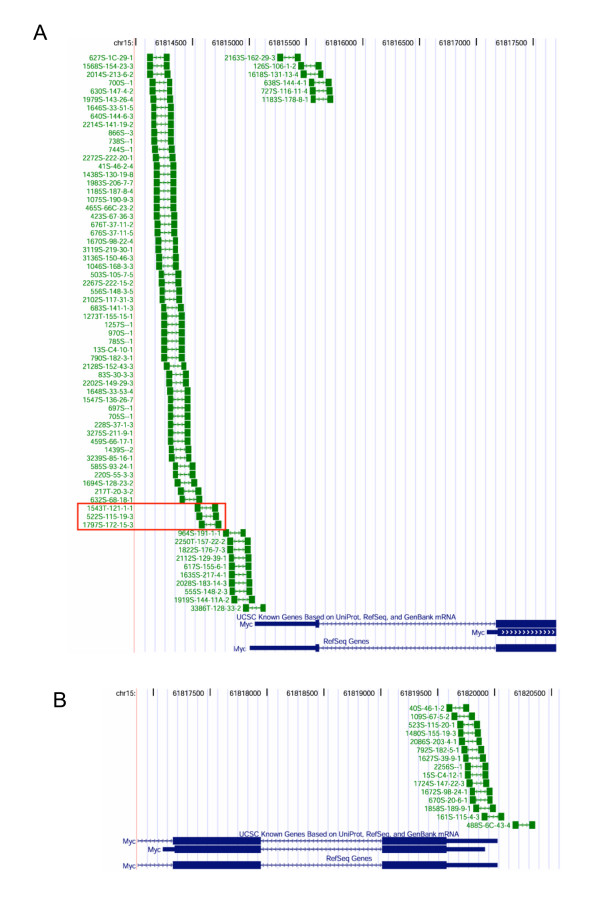
Higher magnification of the **(A) **5' flanking (upstream of exon 1), and **(B) **3' flanking region of *Myc*. The handle bars in green represent the retroviral insertions; arrows in the line within the bars denote direction of provirus transcription. Proviruses boxed in red are in the same orientation as the *Myc *gene (from left to right), opposite from the rest. Proviruses are in the same orientation as the *Myc *gene.

When a genomic region is gene-rich, it is not always straightforward to identify the target gene of insertional mutagenesis. In the past, it has been assumed that the retroviral enhancer can act over a distance of 200 kb in either direction, but without "leapfrogging" a gene promoter. With this assumption, because one of the proximal promoters will always be the retroviral promoter, the orientation of the provirus in regard to the direction of transcription of the gene will be important. Indeed, the two integration clusters into the *Myc *locus are an example of this prediction: the direction of transcription of the provirus upstream of the *Myc *gene always points away from the gene (Fig. 2A; with the exception of the three insertions, boxed in red, which presumably are "promoter insertions," i.e., the transcript is driven by the viral promoter rather than the endogenous promoter). In contrast, the cluster downstream of the *Myc *locus contains proviruses in the same orientation as *Myc *(Fig. 2B). In both cases, this arrangement allows the retroviral 5' enhancer to interact with the *Myc *promoter, although other interpretations are viable (see below). Because of the rule that the retroviral enhancer does not "leapfrog" promoters, but synergizes with the two promoters next to it, the two clusters targeting *Myc *are not expected to directly influence *Pvt1 *transcription, 50 kb downstream.

The criterion of orientation does not hold in an immediately obvious way if a virus integrates into a transcription unit, as it does at the *Pvt1 *locus. In this case, especially as many alternative transcripts have been identified, the exact location of the retrovirus – 5'UTR, 3'UTR, intron, or exon is important. Apart from the retroviral enhancer cooperating with the gene promoter in a conventional manner, the retroviral promoter may override the endogenous promoter, or it may initiate a (truncated) transcript, in addition to truncating or destroying one. If the provirus is located with the UTR, it may also affect mRNA stability, although in that case no preference in proviral orientation would be obvious. If the *Pvt1 *nuclear (primary) transcript encodes miRNAs, we cannot predict the likely consequence of a particular integration – whether the steady-state levels of all or only a few miRNAs change. A low level of *Pvt1 *transcript does not necessarily mean little miRNA product. For example, NIH-3T3 mouse fibroblasts express very little primary RNA of the mir-17-20 cistron, but as much mature mir-17-3p as T cell tumors with retroviral integrations into the primary transcript [23]. This points to the possibility that retroviral insertions do not always have to increase the levels of primary transcripts in order to produce more mature product; instead they might make the processing of miRNA from the primary transcript more efficient.

### Overexpression of *Pvt1 *transcript

**Figure 3:**
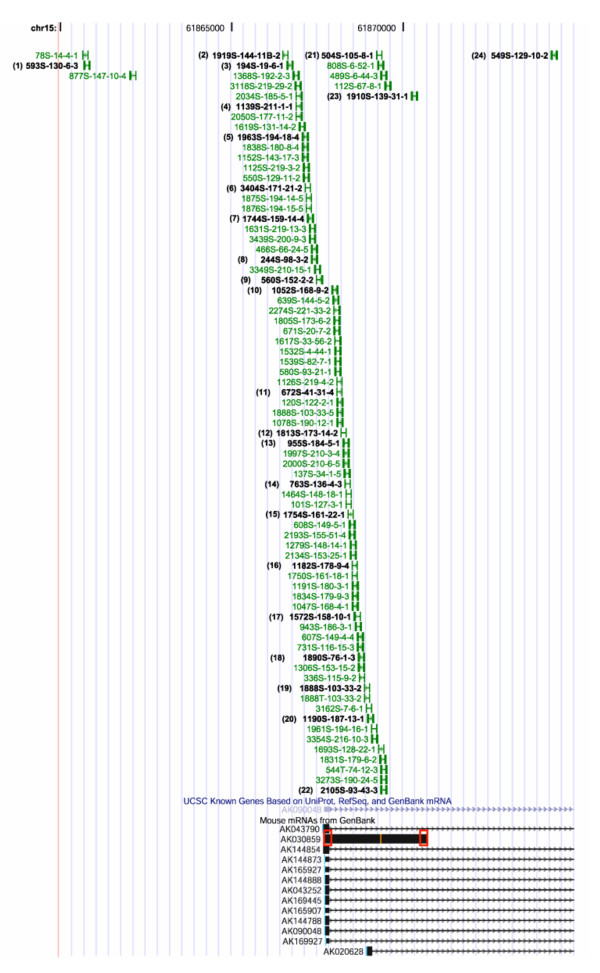
Higher magnification of the area around exon 1 of *Pvt1*, with a main cluster of 78 integrations. Tumors assayed by quantitative PCR (as shown in Figs. 4A to C) are numbered and noted in black text. The locations of the Taqman probes for measuring *Pvt1 *transcript levels are indicated by the red boxes on mRNA AK030859.

**Figure 4:**
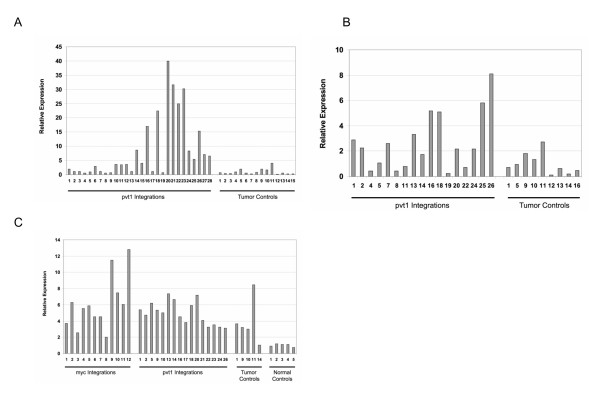
**Expression of the *Pvt1 *and *Myc *transcripts. (A) **Relative expression of exon 1 of *Pvt1*, as measured by quantitative PCR with the 5' primer set depicted in Fig. 3. Tumors numbered 1 through 28 as selected in Fig. 3; control tumors contain integration sites at locations in the genome other than the *Pvt1 *region. **(B) **Relative expression of AK030859 of *Pvt1*, as measured with the 3' primer set depicted in Fig. 3. **(C) **Relative expression of exon 2/3 junction of *Myc*, as measured by quantitative PCR. Tumor controls, tumors with insertions at sites other than the *Myc *or *Pvt1 *locus; normal controls, spleen and thymus cells from mockinjected (no virus) animals.

**Figure 5:**
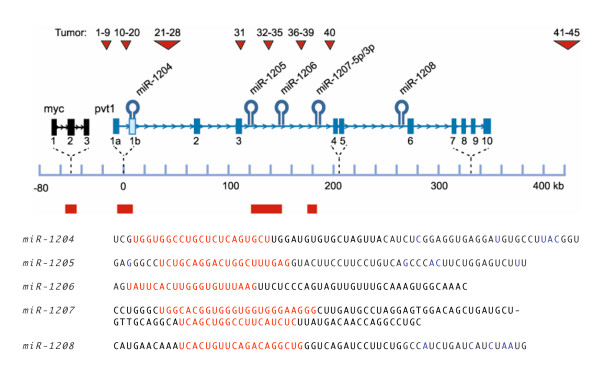
**Schematic representation of the genomic locations of the mouse miRNAs encoded by the *Pvt1 *locus**. Numbers above the red triangles, insertion sites of the tumors tested for miRNA expression; hairpins, location of miRNAs; bars in red below the scale, retroviral integration clusters reported in this study. Below the schematic, genomic sequence of miRNAs and their flanking sequences. The mature miRNA sequences are shown in red.

Fig. 3 shows a higher magnification of the area around exon 1 of *Pvt1*, where a main cluster of 78 integrations is located. Because a plurality of the *Pvt1 *integrations clustered around exon 1, we determined the expression levels of that exon (exon 1a) in various tumors by quantitative PCR, using a primer set that covered the 5' end of this exon (see boxed area in transcript AK030859 depicted in Fig. 3; the 5' end of the exon representing AK030859 is shared with exon 1 of the reference sequence AK090048). Of the tumors with integrations shown in Fig. 3, the designations of tumors we selected are shown in bold face type, and are numbered (1) through (28) (only tumors 1 through 24 are shown in Fig. 3; the integration sites of these tumors, and all other tumors studied here, along with the relative transcription orientation of the proviruses, are given in Table 1). As compared to control tumors, which have no integration into the *Pvt1 *locus, most tumors with the integrations selected in Fig. 3 overexpressed the *Pvt1 *transcript, up to 40-fold (Fig. 4A). Tumors 10 through 28, with insertions starting right at the 3' boundary of the first exon, mostly overexpress *Pvt1 *with a few exceptions (tumors 13, 17, and 19). We have noticed that the direction of transcription of the proviruses in tumors 13 and 19 is opposite of all the others in that group (see above for discussion of provirus transcriptional orientation). Tumors 1 through 9, with insertions located 5' to exon 1a, express *Pvt1 *at levels similar to the control tumors (the control tumors are not listed in Table 1). They possibly overexpress transcripts starting with exon 1b (Fig. 5), although we have not tested this assumption.

**Table 1:** Integration sites of tumors assayed for *Pvt1 *exon 1 transcript, and for *Pvt1*-encoded miRNA expression

**#**	**Tumor**	**Location**	**Orientation**
1	593S	chr15:61860693	T+G+
2	1919S	chr15:61866476	T+G+
3	194S	chr15:61866608	T+G+
4	1139S	chr15:61866868	T+G+
5	1963S	chr15:61867056	T+G+
6	3404S	chr15:61867131	T+G+
7	1744S	chr15:61867205	T+G+
8	244S	chr15:61867333	T+G+
9	560S	chr15:61867464	T+G+
10	1052S	chr15:61867915	G+T+
11	672S	chr15:61868051	G+T+
12	1813S	chr15:61868173	G+T+
13	955S	chr15:61868438	G+T-
14	763S	chr15:61868296	G+T+
15	1754S	chr15:61868379	G+T+
16	1182S	chr15:61868500	G+T+
17	1572S	chr15:61868585	G+T+
18	1890S	chr15:61868694	G+T+
19	1888S	chr15:61869059	G+T-
20	1190S	chr15:61868969	G+T+
21	504S	chr15:61869211	G+T+
22	2105S	chr15:61869357	G+T+
23	1910S	chr15:61870240	G+T+
24	549S	chr15:61874317	G+T+
25	3005S	chr15:61884560	G+T+
26	1437S	chr15:61893983	G+T+
27	455S	chr15:61909406	G+T+
28	2262S	chr15:61922370	G+T+
31	2062T	chr15:61988152	G+T+
32	1784S	chr15:61995200	G+T+
33	1551S	chr15:61996855	G+T+
34	3269T	chr15:61997744	G+T+
35	1309S	chr15:61998706	G+T+
36	1719S	chr15:62041370	G+T-
37	652T	chr15:62041573	G+T+
38	3155S	chr15:62042340	G+T+
39	133S	chr15:62043246	G+T+
40	1820S	chr15:62062642	G+T+
41	1187S	chr15:62285362	G+T+
42	1938T	chr15:62286594	G+T+
43	674S	chr15:62287322	G+T+
44	1907S	chr15:62288987	G+T+
45	1717T	chr15:62290852	G+T+
46	1959S	chr15:62524605	G+T+
C2	1855S	no *Pvt1 *integration site	
C3	3252T	no *Pvt1 *integration site	
C4	3413T	no *Pvt1 *integration site	
C5	3421S	no *Pvt1 *integration site	
C6	1463S	no *Pvt1 *integration site	
C7	1967S	no *Pvt1 *integration site	
C8	14845S	no *Pvt1 *integration site	
C9	1278S	no *Pvt1 *integration site	
C10	1065S	no *Pvt1 *integration site	
*Transcript:*	AK030859	chr15:61867667–61870735	G+

Sites as defined by mm8 version of genome. Orientation, either "+" or "-", of the proviral insert (tag, denoted as T) relative to the direction of the genome display, and to the *Pvt1 *transcript ("gene", denoted as G, here always G+).

Because transcript AK030859 seems to represent a (less frequent) alternative splice product of the putative nuclear transcript, we also performed qPCR analyses with a primer set covering the 3' end of AK030859 (see right boxed area in Fig. 3). In these analyses, tumors with insertions at the *Pvt1 *locus on average expressed more AK030859 sequences than the control tumors, (Fig. 4B) (the control tumors are not listed in Table 1).

### Most T lymphomas express *Myc*, regardless of the location of retroviral integration sites

It is possible that the common integration site at the *Pvt1 *locus is not actually due to selection for tumorigenesis via *Pvt1*, but to preferred (yet unknown) integration sequences at this locus. In this view, the increased *Pvt1 *expression would be of no biological consequence, but the insertions actually would increase *Myc *expression directly. We thus investigated *Myc *expression in tumors with insertions at the *Myc *and *Pvt1 *locus, respectively, and compared them to tumors without insertions at either of these loci; and to normal spleen cells or thymocytes from mock infected (i. e., no virus) mice. Clearly, the normal cell controls expressed less *Myc *than the tumors (Fig. 4C). But by and large, there was not much difference in *Myc *expression among the tumors, whether they had an insertion into the *Myc *locus, the *Pvt1 *locus, or no such insertion (Fig. 4C). Thus the SL3-3 induced T lymphomas generally have elevated *Myc *expression, no matter by which insertion that is accomplished, and there is no obvious correlation between location of insert into the *Pvt1 *locus and *Myc *expression.

It is surprising that although only 6% of the T lymphomas have insertions directly into the *Myc *locus, almost all T lymphomas overexpress *Myc *as compared to normal splenocytes and thymocytes, whether there are insertions into the *Myc *locus, *Pvt1 *locus, or into an unknown site. This fact could be taken as an indication that retroviral integrations are capricious and not always the driving force of tumorigenesis. However, we interpret these data to mean that there may be a requirement for MLV induced T lymphomas in BALB/c mice to overexpress *Myc*, regardless of how this is achieved.

### Identity and expression of miRNAs encoded within the *Pvt1 *region

Although at the time of manuscript preparation no miRNAs were listed in the miRNA registry of the The Wellcome Trust Sanger Institute [31,32] that map to the *Pvt1 *locus, the expressed sequence tag pattern indicated the possibility that *Pvt1 *does encode miRNAs. Indeed, using previously described algorithms that use sequence conservation of putative seed sequences and secondary structural properties of the putative miRNA hairpin structures, *Pvt1*-based miRNA candidates in human, chimpanzee, canine, mouse and rat have been identified [28], and confirmed experimentally in human and mouse [28]. The human miRs have recently been given designations by the Sanger miRBase, and we have adopted the analogous nomenclature for the mouse miRs. Fig. 5 shows the genomic sequences of mouse *Pvt1 *associated miRNAs and their flanking sequences in mouse; the miRNAs are called *mmu-mir-1204, mmu-mir-1205 mmu-mir-1206, mmu-mir-1207-5p, mmu-mir-1207-3p*, and *mmu-mir-1208*. Because in the following, we are only dealing with mouse sequences, we will omit the pre-fix "mmu." The mature miRNA sequences are shown in red. Above the sequences, their relative genomic locations, on chromosome 15, are shown. With *mir-1204 *closest to the *Myc *locus, at a distance of approximately 50 kb, and *miR-1208 *furthest away (305 kb), the pvt-1 primary RNA, if a single transcript, spans at least 255 kb. The exact genomic locations of the Pvt1-encoded miRNA sequences are given in Table 2.

**Table 2:** Genomic locations of the mouse Pvt1-encoded miRNA sequences on chromosome 15, as given by the mm8 and mm9 genome versions.

	miR-1204	miR-1205	miR-1206	miR-1207-5p	miR-1207-3p	miR-1208
mm8	61,869, 066	61,988,887	62,017,747	62,053,091	62,053,155	62,130,913
	61,869, 086	61,988,906	62,017,766	62,053,114	62,053,172	62,130,931
mm9	61,870, 955	61,990,776	62,019,636	62,054,980	62,055,044	62,132,802
	61,870, 975	61,990,795	62,019,655	62,055,003	62,055,061	62,132,820

To determine if the retroviral integrations altered expression of these *Pvt1 *associated miRNAs, we measured the expression of the mature species of the five miRNAs by qRT-PCR using a stem-loop RT primer specific for each miRNA [24,33], in tumors with and without *Pvt1 *insertions (Table 3). For standardization, we compared them to known concentrations of synthesized miRNAs of the relevant sequence. While we could detect a signal for *miR-1206 *only in one tumor, we did find expression of *miR-1204*, *miR-1205*, *miR-1207-5p, miR-1207-3p *and *miR-1208*, albeit at quite different levels. On average, *mir-1204 *was most pronounced as it was expressed nearly four times more in tumors with *Pvt1 *inserts than in the control tumors (Table 3; Δμ 0.05) – irrespective of the site of retroviral integration within the Pvt1 locus. Because thymocytes and spleen cells represent a mixture of many cells, one cannot directly compare these cells with the tumor cells. Nevertheless, we note that the expression level in the tumors with *Pvt1 *integrations was not significantly different from thymocytes and non-stimulated spleen cells. It therefore seems as if this miRNA is required for cell survival. The relatively modest overexpression in tumors with insertions into the *Pvt1 *locus may be a consequence of the retroviral enhancer, but the tumorigenicity of the provirus may be mediated by the persistence of miRNA expression rather than by its overexpression.

**Table 3:** QPCR measuring mmu-miRNAs encoded by *Pvt1*.

**Tumor #**	**miR-1204**	**miR-1205**	**miR-1206**	**miR-1207-5p**	**miR-1207-3p**	**miR-1208**
14	34.54	44.58	BT	29.03	ND	38.96
16	36.35	44.34	36.09	30.39	ND	39.42
20	34.06	42.24	BT	27.92	ND	38.78
21	34.6	39.73	BT	28.39	ND	39.63
22	33.06	41.13	BT	29.6	ND	37.3
23	35.46	42.13	BT	30.07	ND	39.06
31	35.2	39.56	BT	28.43	34.26	38.57
32	35.87	38.65	BT	30.07	34.93	38.9
34	33.3	38.59	BT	28.86	35.3	41.44
35	33.72	42.19	BT	29.91	34.3	ND
36	35.83	40.16	BT	30.85	35.47	39.5
39	33.16	42.44	BT	30.89	35.52	41.3
40	35.05	38.94	BT	31.14	40.16	45.52
41	34.95	43.62	BT	31.15	37.2	40.39
42	34.2	41.75	BT	29.44	35.64	40
43	33.92	40.13	BT	29.45	35.91	40.73
44	36.9	39.98	BT	31.34	36.45	42.1
45	33.36	41.07	BT	27.25	34.72	38.81
46	35.04	42.21	BT	28.93	36.1	41.29

**Average ± STD**	**34.66 ± 1.12**	**41.23 ± 1.82**	**ND**	**29.64 ± 1.17**	**35.84 ± 1.54**	**40.09 ± 1.83**

C2	38.02	42.23	BT	30.28	36.93	40.53
C3	33.53	41.93	BT	26.98	34.6	39.36
C4	36.19	41.1	BT	29.3	35.45	39.62
C5	36.03	45.22	BT	30.23	ND	39.23
C6.2	ND	37.35	ND	ND	40.31	40.36
C6.3	36.9	42.82	BT	31.77	38.64	41.73
C7	37.08	BT	BT	32.11	ND	41.11
C8	36.51	43.14	BT	30.89	ND	39.55
C9	37.15	44.73	BT	30.86	ND	40.32
C 10	36.8	45.2	BT	30.4	ND	39.26

**Average ± STD**	**36.47 ± 1.25**	**42.64 ± 2.47**	**BT**	**30.31 ± 1.51**	**37.19 ± 2.33**	**40.11 ± 0.85**

thymus	35.01	34.41	BT	33.06	38.17	43.4
spleen	34.05	40.3	BT	31.03	36.4	41.97
Reject H0	yes	no		no	no	no

Numbers in the first column correspond to the tumour numbers in the Table 1; other columns represent the cycle numbers needed to reach a predetermined threshold. BT, below threshold; ND, not done; STD, standard deviation. A cycle number of 30 corresponds to approximately 3000 molecules per cell.

Although *miR-1205 *and *miR-1208 *gave clear signals, the threshold was only reached after 40 cycles, making the significance of these miRNAs in tumorigenesis less clear. However, in three tumors (#31, #32, #34) with integrations close to its genomic position, *miR-1205 *is expressed more than in other tumors; and the expression level of *miR-1205 *in thymus (34 cycles to reach threshold; Table 3) makes it likely that *miR-1205 *plays a role in normal cell differentiation. In most of the tumors, we did not find *miR-1206 *expression; although precursor RNA was increased in the mouse myeloma MOPC104E [28], we did not find the mature miRNA expressed (not shown). In fact, there was also no expression in thymocytes and spleen cells, but tumor 16 gave a clear and reproducible signal. Since this tumor does not differ in its integration site or proviral transcriptional orientation from other tumors with insertions in this region, we think that the *miR-1206 *expression is not mediated by the provirus. Rather it may be the effect of another mutational event, which in myelomas is more frequent. The level of *mmu-miR-1207-5p *was relatively low in thymus; but the levels of *miR-1207-5p *and *miR-1207-3p *in tumors with and without integrations into the *Pvt1 *locus did not differ much, and thus we cannot correlate expression of these miRs with an oncogenic event. In all the tumors, it is possible that the other allele (with no proviral integration) contributes to the miRNA levels, which may mask differences.

Overall we can conclude that except perhaps for *miR-1206*, the other *Pvt1 *encoded miRNAs are expressed in T-lymphocytes. However, we have not yet performed a detailed analysis of the consequences of the various proviral integrations sites. We can assume that the exon 1 overexpressing tumors end their transcripts with the retroviral termination site and poly A tail, which would exclude all the downstream miRNAs. However, the 3' retroviral promoter may also restart a transcript, as has been discussed for integrations into the Notch1 locus [34]. An indication for this is the fact that the qPCR primers covering the the 3' end of the intron-less transcript AK030859, also measured increased expression levels in tumors with insertions between the DNA segments of probe sets 1 and 2. At any rate, we feel justified in concluding that except perhaps for *miR-1208*, all other *Pvt1 *encoded miRNAs do exist, and that it is likely that murine *mir-1204 *is oncogenic in T lymphomas when constitutively expressed.

### Co-mutation analysis

**Figure 6:**
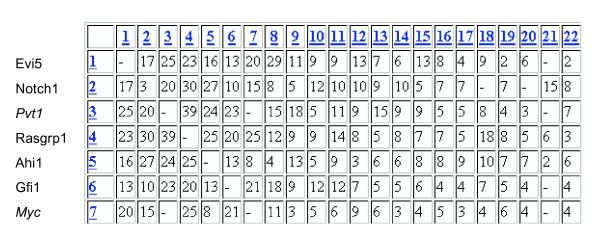
**Matrix for co-mutation analysis**. Only a partial view is given. The numbers (color blue, underlined) represent the oncogenes/tumor suppressor genes detected in the T lymphoma screen, in the order of their incidence, horizontally and vertically. The numbers in the boxes at the intersections (color black) indicate the number of tumors the cancer genes were found in the same tumor.

It is well established that tumorigenesis is the result of accumulating several cooperating mutations that drive relentless proliferation and aid in metastases. Co-mutation analyses, where one oncogenic event is fixed by means of a transgene in the mouse to be infected with retrovirus, were very successful in identifying cooperating oncogenes, for example with *Myc *[14], or with p27Kip1 loss [19]. Without fixing any event by a transgene, viral insertional mutagenesis, though perhaps not providing all the mutations necessary for a full-blown tumor, follows the multistep scenario of tumorigenesis. Although in general the superinfection barrier largely prevents multiple proviral integrations within the same cell, re-infection does happen over time. Because it is a rare event, such cells are selected over the others only when these integrations also give a growth advantage. As a consequence, in general, most viral insertions ("co-mutations") in a single tumor are thought to be causative in its formation. With the caveats of potential passenger mutations and potential oligoclonality of tumors, co-mutation analysis may be a powerful way to find cooperating signaling pathways in tumorigenesis. For this analysis, the following two rules can be stated: (i) genes that are co-mutated in a single cancer cell represent different pathways that cooperate during carcinogenesis; and (ii) genes within the same pathway are never co-mutated. These rules assume "linear," non-branched pathways, which is a gross oversimplification. They also assume that it does not help to turn on a pathway (twice) by two integrations rather than one, but, of course, increased signal strength may indeed help tumorigenesis. For example, an obvious exception is Notch1, for which two mutations have been shown to lead to more aggressive growth than just one [35] – a fact that is reflected by our finding of three double mutants (Fig. 6; and unpublished), with mutations in the same two domains that are also co-mutated in patients. Nevertheless, if in a large sample set, one never finds two genes co-mutated, it seems fairly safe to assume that they are in the same pathway.

Fig. 6 shows a matrix for co-mutation analysis, here focusing on *Pvt1*. The numbers (color blue, underlined) represent the frequency of tagged oncogenes/tumor suppressor genes detected in the T lymphoma screen, in the order of their incidence, horizontally and vertically. The numbers in the boxes at the intersections (color black) indicate the number of tumors the cancer genes were found in the same tumor. In this matrix, *Pvt1 *is represented by number 3, i.e., it is the third most frequent cancer locus in mouse T lymphomas mutated by MLV. The other high frequency tagged genes (1, 2, 4, 5, 6 and 7) are Evi5, Notch1, Rasgrp1, Ahi1, Gfi1 and *Myc*, respectively. Because they are found together with *Pvt1 *in a number of tumors, these genes are all co-mutations, except for *Myc *– there are no tumors with insertions to both *Myc *and *Pvt1 *loci. By the logic above, this apparently places *Pvt1 *and *Myc *into the same pathway, although from this analysis it cannot be determined which one of the genes is upstream. Another indication for the two genes sharing a pathway comes from the fact that they have the same co-mutations (Fig. 6); and that they both do not co-mutate with gene 21, which thus ought to be in the same pathway as well.

With 11% of the 2199 T lymphomas studied having insertions within it, the pathway pvt is part of what seems to be one of the most important regulators of T lymphomagenesis in the BALB/c mouse strain, and, by extension, perhaps also in Burkitt's lymphoma and in mouse plasmacytomas, as these are clearly driven by the translocations involving the *Myc *and *Pvt1 *loci. If so, it seems peculiar that in our screen with Akv, which induces B cell lymphomas in NMRI mice, only one out of 1798 tags were in the *Myc *locus, and none in the *Pvt1 *locus. Similarly, among the resulting 24 tumors analyzed by Lovmand et al. [15], only one tumor, containing an Akv variant, harbored a clonal proviral integration in the c-*Myc *locus. Because most cells, including B cells, express the Akv receptor, the reason for this may lie in the differentiation stage of the infected cell.

## Conclusion

Part of the complexity in determining the functional significance of the *Pvt1 *locus stems from the fact that *Pvt1 *is closely linked to the *Myc *locus. Translocations directly into the *Myc *locus change expression levels of *Myc*, and thus easily explain their contribution to oncogenesis; but the breakpoints of variant translocations into the *Pvt1 *locus extend up to 400 kb downstream of *Myc*, and they also have been thought to cause overexpression of *Myc *as well. Because the *Pvt1 *transcript encodes no protein, the effect on *Myc *was thought to be direct, and, therefore, long range [4]. On the other hand, the multiple myeloma cases with translocations in the *PVT1 *locus without immunoglobulin gene translocation would argue for this locus to be oncogenic in its own right, as do the retroviral integrations into this locus.

In this paper, we present a large number of tumors with retroviral integrations into the *Pvt1 *locus, which thus can be regarded as oncogenic, particularly as these integration events are associated with overexpression of *Pvt1 *transcripts. We also confirm that the *Pvt1 *locus encodes miRNAs, and that retroviral insertion can lead to altered expression of at least one of these miRNAs. From the co-mutation analysis, we also conclude that *Pvt1 *and *Myc *are likely in the same pathway; this may mean that any of the miRNAs directly determine *Myc *transcript levels by siRNA-type mediated degradation; or, because there is no clear binding site for any of these miRNAs in the 3'UTR of *Myc*, more likely by regulating the translation of upstream factors that activate *Myc*. Consistent with this hypothesis, over-expression of *mir-1204 *in mouse pre-B cells, but not in pro-B cells, appears to increase *Myc *expression [28] – apparently in a cell type and/or stage specific fashion. Conversely, *Myc *may also regulate the levels of *Pvt1 *encoded miRNAs. Which of these alternatives is the case may be decided once the targets of the miRNAs are known.

## Methods

### Retroviral induction of tumors of mice

BOSC23 retroviral packaging cells were transfected with plasmids encoding the complete SL3-3 provirus. Viral particles from culture supernatant were injected intraperitoneally into newborn (<3 days) BALB/c mice. The fathers of the injected mice were also mutagenized by ethyl-nitroso-urea as part of another study [36]. Mice were monitored everyday for general sickness as well as tumor development. When sickness or tumors of defined size were discovered, mice were euthanized and tumors of the spleen and thymus were removed and frozen at -80°C.

### Identification of provirus integration sites

The genomic locations of the proviral integrations were determined using the splinkerette-based PCR method [37]. This method recovers genomic DNA directly flanking the 5' LTR of the integrated provirus. Genomic DNA was isolated from tumors using the DNeasy Tissue kit (Qiagen) and digested using restriction enzymes BstYI or NspI. A double-stranded splinkerette adapter molecule [38] containing the appropriate restriction site was ligated to the digested genomic DNA using the Quick Ligation kit (New England Biolabs). These ligation products were then digested with EcoRV to prevent subsequent amplification of internal viral fragments. The resulting mixture was purified using QIAquick PCR purification kits (Qiagen), and subject to three rounds of PCR using nested PCR primers that had homology to the adapter DNA and to the 5' LTR sequence of the SL3-3 virus. After resolving the PCR products by gel electrophoresis, the desired bands were purified using QIAquick Gel Extraction kits (Qiagen) and subject to standard DNA sequencing.

### Quantitative PCR of primary RNA transcripts

Total RNA was extracted from frozen mouse spleen and thymus tumor samples using the RNeasy Mini Kit (Qiagen). All RNA samples were treated with rDNase (Ambion) prior to reverse transcription. 500 ng RNA from each tumor sample was reverse transcribed with random hexamers using the SuperScript First-Strand Synthesis System III (Invitrogen). qPCR was conducted on the Stratagene MX3000P using Brilliant SYBR Green qPCR Master Mix (Stratagene). SYBR qPCR primers were designed using Beacon Designer 5.0 from Premier Biosoft and ordered from Integrated DNA Technologies. Beta-actin (ACTB) served as an endogenous control gene for all SYBR qPCR runs. qPCR primers were as follows: ACTB: 5'-TTCCAGCCTTCCTTCTTG-3', 5'-GGAGCCAGAGCAGTAATC-3'; *Pvt1*-exon1: 5'-(GAGCACAT)GGACCCACTG-3' (it contains 8 bp of genomic sequence before the start of AK090048 exon1, genomic part in parenthesis); 5'-GCTGCCAACATCCTTTCC-3'; AK030859 (3'end): 5'-GGCACAAGAGAACCAAGTCC-3', 5'-CGCTTATCCTCCTGCTTCAAC-3'; and *Myc*-ExJ2-3: 5'-GACACCGCCCACCACCAG-3', 5'-GCCCGACTCCGACCTCTTG-3'.

The qPCR reaction mixture contained 150 nM (final concentration) of each primer and the appropriate dilution of cDNA for each target studied in a final qPCR reaction volume of 25 μl. PCR cycling was as follows: 95°C 10 min; 40 cycles of 95°C 30 sec, Ta (annealing) of 55 to 60°C 60 sec, 72°C 30–45 seconds; followed by a denaturation cycle of 95°C 60 sec, 55°C 30 sec, 95°C 30 sec. Tumor samples containing no integration sites in the region of interest were used as control tumors. Relative expression values (2^-ΔΔCt^) were calculated using control tumor 1 as a calibrating sample. All relative expression values were then normalized to set the average of the tumor controls to a value of 1 for each target.

### Quantitative PCR of miRNAs

MiRNAs and low molecular weight RNAs were isolated from frozen mouse tumor tissue using the Purelink miRNA Isolation Kit (Invitrogen). The mature species of the miRNAs were measured by RT-qPCR using a stem-loop RT primer specific for each miRNA [33] in the cells listed, in triplicates. Accordingly, 50 ng of each tumor miRNA preparation was reverse transcribed with the SuperScript III First-Strand Synthesis System for RT-PCR using the following stem loop RT primers (50 nM final concentration) 5'-GTCGTATCCAGTGCAGGGTCCGAGGTATTCGCACTGGATACGACAGCACT-3'(mmu-mir-1204), 5'-GTCGTATCCAGTGCAGGGTCCGAGGTATTCGCACTGGATACGACCTCAAA-3'(mmu-mir-1205), 5'-GTCGTATCCAGTGCAGGGTCCGAGGTATTCGCACTGGATACGACACTTAA-3' (mmu-mir-1206), 5'-GTCGTATCCAGTGCAGGGTCCGAGGTATTCGCACTGGATACGACCCCTTC-3'(mmu-mir-1207-5p), 5'-GTCGTATCCAGTGCAGGGTCCGAGGTATTCGCACTGGATACGACGAGATG-3'(mmu-mir-1207-3p) and 5'-GTCGTATCCAGTGCAGGGTCCGAGGTATTCGCACTGGATACGACCCAGCC-3'(mmu-mir-1208). The reverse transcription reactions were diluted 1:5 and 5 μl of these dilutions were used in the 25 μl qPCR reactions. The annealing step was 50°C for 60s. The qPCR probes and primers were as follows: mmu-mir-1204: 5'-GCGGTGGTGGCCTGCTCT-3', 5'-GTGCAGGGTCCGAGGT-3', 5'-[56-FAM]-CACTGGATACGACAGCACTG-[36-TAMSp]-3'; mmu-mir-1205: 5'-GGCGTCTGCAGGACTGG-3', 5'-GTGCAGGGTCCGAGGT-3', 5'-[56-FAM]-CACTGGATACGACCTCAAAG-[36-TAMSp]-3', mmu-mir-1206: 5'-TTGCGGTATTCACTTGGG-3', 5'-GTGCAGGGTCCGAGGT-3', 5'-[56-FAM]-CACTGGATACGACACTTAAACA-[36-TAMSp]-3'; mmu-mir-1207-5p: 5'-TGCTGGCACGGTGGGTG-3', 5'-GTGCAGGGTCCGAGGT-3', 5'-[56-FAM]-CACTGGATACGACCCCTTCC-[36-TAMSp]-3'; mmu-mir-1207-3p: 5'-TGTCTGTCAGCTGGCCT-3', 5'-GTGCAGGGTCCGAGGT-3', 5'-[56-FAM]-CACTGGATACGACGAGATGA-[36-TAMSp]-3'; mmu-mir-1208: 5'-CCGGTCACTGTTCAGAC-3', 5'-GTGCAGGGTCCGAGGT-3', 5' [56-FAM]-CACTGGATACGACCCAGCCT-[36-TAMSp]-3'.

Synthetic RNA oligos (IDT) were used to generate a calibration curve for each miRNA; for mmu-mir-1204, 5'-UGGUGGCCUGCUCUCAGUGCU-3'; mmu-mir-1205, 5'-UCUGCAGGACUGGCUUUGAG-3'; mmu-mir-1206, 5'-UAUUCACUUGGGUGUUUAAGU-3'; mmu-mir-1207-5p, 5'-UGGCACGGUGGGUGGGAAGGG-3'; mmu-mir-1207-3p, 5'-UCAGCUGGCCUUCAUCUC3'-3'; and mmu-mir-1208, 5'-UCACUGUUCAGACAGGCUGG-3'. Amplification efficiencies of the calibration curves for the 6 mmu-mirs were at least 70%. Concentrations of the mature species were calculated using the calibration curves and then normalized by the average of the control tumors, to calculate relative expression levels.

## Authors' contributions

GBB designed and carried out the quantitative PCR experiments for the miRNAs; AML designed and carried out the quantitative PCR experiments for the primary RNA transcripts and helped in the drafting of the manuscript; KH and NJC previously identified the mouse miRNA-PVT sequences, and KH was intimately involved in discussions about the ongoing work and helped with the manuscript; BBW carried out the tag recovery and identification; BBW and MW planned and directed the execution of the retroviral screen and the design of the study, and MW wrote the manuscript.
